# Experimental Investigation on Choosing a Proper Sensor System for Guided Waves to Check the Integrity of Seven-Wire Steel Strands

**DOI:** 10.3390/s20185025

**Published:** 2020-09-04

**Authors:** Edison Z.Y. Hou, Javad Rostami, Kim Ming Ng, Peter W. Tse

**Affiliations:** Smart Engineering Asset Management Laboratory (SEAM) and the Croucher Optical Nondestructive Testing and Quality Inspection Laboratory (CNDT), Department of Systems Engineering & Engineering Management, City University of Hong Kong, Hong Kong, China; edisonhou2-c@my.cityu.edu.hk (E.Z.H.); jrostami2-c@my.cityu.edu.hk (J.R.); kimmingng2-c@my.cityu.edu.hk (K.M.N.)

**Keywords:** structural health monitoring, non-destructive testing, longitudinal ultrasonic guided waves, piezoelectric transducer, magnetostrictive sensors, laser-generated ultrasonic guided waves

## Abstract

Multiple wire twisted steel strands are commonly used to hoist elevators, concrete structures, etc. Due to frequent and long-time usage, the steel strands are subjected to corrosion, overloads, and aging, making strands may fail unexpectedly. Hence, the health monitoring of steel strands becomes more important to avoid the sudden collapse of hoisting structures. Guided waves (GW) inspection methods have become favorable in recent years due to its long-distance transmission and stability of evaluation in the area of structural health monitoring (SHM) and Non-Destructive Testing (NDT). Many researchers have reported different GW methods to detect different types of defects that occurred in steel strands. However, researchers rarely carry out comparative studies to investigate the effectiveness of each method or system in monitoring the health state of steel strands. This article reports some vital observations revealed from conducting experiments by using contact and noncontact methods, which include three different popular types of GW sensors and methods during their applications in surface-type defect detection. The proper selection of sensors systems has been identified through the present study. The result of the present study is believed to be useful guidance for selecting appropriate GW methods and sensor systems to monitor the integrity of the steel strand and thereby ensure the safety of the hoisted structures.

## 1. Introduction

The rapid development of infrastructure and technological innovations has encouraged many researchers to come up with novel ideas to inspect and monitor different structures. The appropriate inspection is determined based on the different principles of complex structures used in the engineering field. Among these complex structures, the steel strand/cable is one of the necessities in the engineering field, and it is difficult to easily replace it in any engineering problem. Steel strands are obviously highly needed in construction-related projects. These types of steel cables or wires, if not taken care of, can lead to serious accidents and disasters. In the United States, incidents involving elevators lead to 31 deaths and serious injuries to 17,000 people each year. Additionally, in 2013, all four cables of a lift (up to 12 people) broke at the same time in Hong Kong, resulting in seven injuries, of which three were critically injured [1,2]. Therefore, proper testing of its quality has become a prerequisite for ensuring the quality of construction projects and the safety of life and property.

There are many methods for inspecting the health condition of steel strands, such as methods based on Magnetic Flux Leakage (MFL) [3], optical methods [4], Acoustic Emission (AE) [5], and Pulsed Eddy Current. The MFL method is very effective in detecting small defects and cable breaks; however, it is a point-to-point method that could be somewhat time-consuming for long-range inspection. Optical-based methods cannot detect underground defects [4]. The AE technique is a passive method and is employed for continuous health monitoring of cables, but it cannot capture existing failure [5]. Pulsed eddy current is capable of identification of hidden corrosion, but it cannot be applied to non-conductive materials. Moreover, these methods may hardly be used in the anchoring area detection. For the damage detection in the anchorage zone, the ultrasonic guided wave (UGW) has proven to be an effective method to some extent and thus it has gained more and more interests. For example, Yong-dong Pan et al. [6] used high order longitudinal guided waves in the anchorage area of the stayed cable. Generally, an effective inspection method must be able to detect and identify defects not only to anchorage zone at an early stage so that appropriate maintenance measures can be implemented. UGW is indeed an effective method for remote inspection of plate-like structures and spiral waveguides such as steel strands [7,8].

Several researchers have conducted some detailed analysis of UGW in steel strands. For example, O. Onipede, JR [9] proposed a method to determine the vibration frequency and the corresponding modal mode of twisted bars. Hegeon Kwun [10] found that in the unloaded state, the wave propagation characteristics in the strands were the same as those seen in individual wires comprising the strand, namely, straight center wire and helical wires. Salim Chaki et al. [11] performed acoustoelastic calibration tests and built a small-model of seven-wire steel strand anchorage block to verify the method dealing with a non-destructive procedure for evaluating the stress level. Renaldas Raisutis et al. [12] studied the propagation of UGW along composite multi-wire ropes under various types of acoustic contacts between neighboring wires and the plastic core. For steel strand/cable defect detection, Rizzo, P. [13] employed wafer piezoelectric sensors to address an experimental investigation on the ultrasonic wave propagation in seven-wire strands loaded at different stress levels, and the lowest-order longitudinal mode is studied at different levels of load. Bartoli et al. [14] used low-profile piezoelectric sensors to probe the individual wires comprising the strand and used semi-analytical finite element analysis to predict modal and forced wave solutions as a function of the applied prestress level. Xinjun Wu et al. [15] found that energy exchange among the wires decreases along with the same propagating distance as the frequency increases. For other aspects, different inspection methods have also been developed because of the tremendous inspecting demands for other structures of such a kind and their accessories. For example, Gianpiero Trane et al. [16] presented the proprietary design and implementation of a piezoelectric transducer (PZT) sensor for the transmission and reception of guided waves that use a multiple-wire AWG12 cable, commonly used in electric domestic and industrial applications, as a communication channel. Marina Adel Shokry et al. [17] recently proposed a new criterion for the detection and localization of four types of defects that might occur in the cable sheath when a cross-bonding configuration is adopted in which this criterion can be applied at a load current of 25% or greater. Junping, C. et al. [18] have proved the feasibility of using the ultrasonic-guided wave for Non-Destructive Testing (NDT) of the aluminum sheath of the high-voltage (HV) cable.

Some scholars have investigated the guided wave propagation through numerical methods and have developed algorithms that suitable for the field of steel strand inspection [19,20,21,22,23], and some other researchers have reported the use of signal processing methods to analyze UGW signals in strands [24,25,26,27]. For example, Hernandes-Salazar, C.D. et al. [25] successfully used Continuous Wavelet Transform (CWT) to observe the multimode nature of guided waves, which showed that the propagation in the cables was mainly localized on the single wires.

Some parts of research achievements utilizing non-destructive detection and other inspection methods or relevant areas for strand/cable inspection have been shown above. However, as of today, there have been very few comparative experimental studies on NDT methods of using UGW for seven-wire twisted steel strand inspection. NDT methods are used to detect defects in steel cables/strands or other types of structures used in engineering. One appropriate method for inspecting this type of structures under certain environmental conditions must be chosen after considering technical issues (inspection ability, detecting stability, etc.) and economic indicators such as cost of human resources, operations, and equipment maintenance. Therefore, the purpose of this research is to promptly propose some practical suggestions on how to choose a suitable NDT method based on UGW to inspect defects in the steel strands under different engineering conditions in a simple way.

The next several parts will be arranged as follows, the second part will be the theoretical background for each type of sensor that we used, and the third part is an experimental investigation with magnetostrictive sensor (MsS), PZT, and laser-generated GW, followed by a fourth and fifth section, which are the discussion and conclusion, respectively.

## 2. Theoretical Background

### 2.1. Theory of Magnetostrictive Sensor

The principle of MsS is mainly based on the phenomenon of magnetostriction. The medium will be magnetized when placed in a magnetic field. The magnetic induction intensity in the magnetic medium *B* meets Equation (1):

(1) $$B=\mu_{0}\left(H+M\right)$$

where *B* is the magnetic induction intensity, *H* is the magnetic field intensity, *M* is the magnetization intensity, and $\mu_{0}$ is the magnetic conductivity of the vacuum. The relationship between the magnetic field intensity *H* and the magnetization intensity *M* is shown in Equation (2); χ is the magnetic susceptibility:

(2) $$\chi \;=\frac{M}{H}$$

The magnetic medium can be divided into ferromagnetic substance, diamagnetic substance, and paramagnetic substance, according to the relationship between magnetic induction intensity *B* and magnetic field intensity *H*. In terms of the diamagnetic substance, magnetic induction intensity *B* is less than magnetic field intensity *H*. For the paramagnetic substance, magnetic induction intensity *B* is greater than magnetic field intensity *H.* Magnetic induction intensity *B* of the ferromagnetic substance is significantly greater than that of magnetic field intensity *H*. As described above, domain-wall movement and magnetic torque rotation occurs during the magnetizing process. It shows that these will cause deformation of the ferromagnetic material; this is called magnetostriction. The deformation δl*/*l changes as the applied magnetic field increases, which will reach saturation *λ*. The deformation δl*/*l meets Equation (3):

(3) $$\frac{\delta l}{l}=e.cos^{2}\varphi$$

where *e* is the saturation deformation, and *φ* is the included angle between the direction of magnetization and the direction of observation.

When the ferromagnetic material is placed in a time-varying magnetic field, a dimensional change arises in the ferromagnetic material, depending on the field’s magnitude and direction. This is known as the Joule effect or magnetostriction effect. When the ferromagnetic material under a magnetic field is subjected to a change in the stress field, it exhibits a change in its amount of magnetization. The inverse magnetostriction effect is called the Villari effect. These two effects can be expressed as Equation (4), as follows:

(4) $$\left(\frac{\partial \varepsilon }{\partial H}\right)._{\sigma }=\left(\frac{\partial B}{\partial \sigma }\right)._{H}$$

where *ε* and *σ* are the stress and strain of the ferromagnetic material, respectively. In addition to the Joule effect, the Wiedemann effect is another kind of magnetostriction effect that produces shear stress. Longitudinal and torsional wave modes are generated by the Joule effect and the Wiedemann effect, respectively [28].

### 2.2. Theory of Piezoelectric Transducer

Piezoelectric materials were discovered in the 1980s, and according to their different physical structures, piezoelectric materials can be divided into piezoelectric crystals, piezoelectric ceramics, piezoelectric films, piezoelectric polymers, and piezoelectric composites. The piezoelectric transducer is currently widely used in industry due to their high energy and high signal-to-noise ratio (SNR). With the development of GW technology for a long time, scholars around the world have been studying the use of PZT to trigger GW and achieved a lot of breakthrough results.

Piezoelectric sensors use the piezoelectric effect of piezoelectric materials to excite ultrasonic waves; at the same time, based on the inverse piezoelectric effect of piezoelectric materials, piezoelectric sensors can receive ultrasonic waves. The substantial principle of exciting the ultrasonic guided wave in the waveguide structure is to transmit the vibration indirectly or directly to the measured workpiece through couplant or dry coupling. Piezoelectric materials have a special property, the piezoelectric effect, which is of great importance in daily practical applications. It can be classified into two types, the positive piezoelectric effect, and the inverse piezoelectric effect. The positive piezoelectric effect is also called the piezoelectric effect. When the crystal is in the absence of an electric field, its strain makes the internal charge polarized. The specific principle is: when the crystal is subjected to external force, there will be a certain deformation, so that the positive and negative charges inside the piezoelectric element will dynamically shift, and eventually polarized charges of opposite signs will appear on the crystal surface. Through this principle, piezoelectric materials can be made into sensors to determine whether the structure is deformed. On the contrary, the inverse piezoelectric effect is a situation where an electric field is applied to the crystal, and the positive and negative charges inside the piezoelectric element are dynamically shifted. In this process, the interaction of the charges causes its deformation. According to this principle, piezoelectric materials can be made into actuators to limit the geometric changes of the structure.

When studying the vibration state of piezoelectric ceramics in a unique direction (that is, along with the length and thickness directions), the stress-strain relationship can be regarded as meeting the unique condition, and the piezoelectric effect expression can be simplified to the following Equations:

Stress:

(5) $$T_{s}=E_{p}\left(S-d_{31}E_{s}\right)$$

Strain:

(6) $$S=\frac{1}{E_{p}}T_{s}+d_{31}E_{s}$$

Electric displacement:

(7) $$D=d_{31}T_{s}+\varepsilon_{33}^{T}\;E_{s}$$

where in Equations (5)–(7), $T_{s}$ is stress, *S* is the strain, $E_{s}$ is electric field strength, *E_p_* is elastic modulus, *D* is electric displacement, $\varepsilon_{33}^{T}$ is dielectric constant, and *d_31_* is strain constant.

The piezoelectric constitutive equations can be written as being:

(8) $$D_{c}=e^{\sigma_{s}}.E+d_{p}.\sigma_{s}\;\left(\mathrm{direct}\;\mathrm{effect}\right)$$

(9) $$\sigma_{s}\;=-{\left(d_{p}.C^{E}\right)}^{T}.\;E+\;C^{E}.\varepsilon_{s}\;\left(\mathrm{converse}\;\mathrm{effect}\right)$$

where in Equation (8), $D_{c}$ is the charge density vector (3 × 1), $e^{\sigma_{s}}$ is the dielectric permittivity matrix (3 × 3) measured at zero stress, *E* is the electric field vector (3 × 1), $d_{p}$ is the piezoelectric coefficient matrix (3 × 6), and $\sigma_{s}$ is the stress vector (6 × 1). In Equation (9), $C^{E}$ is the elastic stiffness matrix (6 × 6) measured at zero electric fields, $\varepsilon_{s}$ is the strain vector (6 × 1), and the superscript *T* indicates a transposed matrix [29].

**Figure 1:**
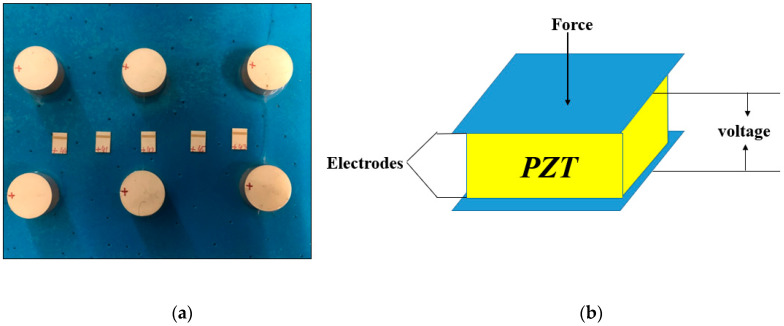
Piezoelectric transducer (PZT): (**a**) Commonly used circular PZT and square PZT; (**b**) The composition of PZT.

The piezoelectric sheet or patch is the most basic element for exciting guided waves. As shown in Figure 1, it is a piezoelectric sheet that vibrates in one direction in ultrasonic guided wave testing. Usually, we divide them into drivers and sensors. Converted into displacement; use the positive piezoelectric effect of the PZT material as a sensor. When the structure is deformed and transferred to the exchanged PZT sheet, it is equivalent to applying an external force or displacement to the PZT sheet. The surface of the deformed P-side T sheet will have a positive and negative offset, which is finally received by the signal acquisition device, thereby acting as a sensor.

According to Snell’s Law, when an ultrasonic wave enters the waveguide medium obliquely, the angle of refraction of the transverse wave and the longitudinal wave changes when they pass through the boundary of the medium due to the different wave speeds. According to this principle, different modes of guided waves in the waveguide medium can be excited. In practical applications, piezoelectric oblique probes are often used to form an array for guided wave excitation.

### 2.3. Theory of Laser-Generated Ultrasound

Laser-generated ultrasound is a commonly used NDT technique for remote and noncontact transduction. It is a combined effect of heat absorption and thermal expansion. When the laser beam strikes the surface of the specimen most of the electromagnet energy is absorbed, and the solid particles are excited as a result. As such, sudden local thermal expansion of the specimen creates a mechanical wave that propagates along and into the whole length of the specimen. The governing transient thermal conduction equation is expressed as follow [30]:

(10) $$\rho C_{v}\frac{\partial T}{\partial t}-\nabla \cdot \left(K\nabla T\right)=Q$$

where ρ is the density, $C_{v}$ is the specific heat capacity, T is the temperature distribution, K is the thermal conductivity coefficient, and Q is the power density. After absorption of the incident laser energy, ultrasonic waves are excited due to the effect of thermal expansion and is expressed as:

(11) $$\mu \nabla^{2}u+\left(\lambda +\mu \right)\nabla \left(\nabla \cdot u\right)-\alpha \left(3\lambda +2\mu \right)\nabla T=\rho \frac{\partial^{2}u}{\partial t^{2}}$$

where u is the transient displacement field, λ and μ are the Lamé constants, and α is the thermal expansion coefficient.

To generate ultrasonic waves at the desired frequency range using a laser, the laser pattern can be modulated spatially. The spatially modulated laser-pattern has to be in the form of the equidistant line array. The element width of the laser pattern determines the wavelength of the generated wave. Therefore, the frequency of the ultrasonic waves can be controlled by adjusting the element width of the laser pattern. In the present study, the Integrated Sagnac Interferometer-based Optical System (SIOS) optical system [31] was used to generate a line-array pattern. The governing equation of the line-array pattern is expressed as:

(12) $$Q=A_{b}I_{0}cos^{2}\left(\frac{\pi }{d}x_{1}\right)T_{1}$$

where $A_{b}$ is the absorptivity of the specimen, $I_{0}$ is the spatial distribution of the laser beam, and $cos^{2}\left(\frac{\pi }{d}x_{1}\right)$ is the interference term which governs the element width of the pattern, d is the element width, and $T_{1}$ is the temporal profile of the laser pulse.

To predict the desired wave mode that would be generated, a constant wavelength line can be drawn on the dispersion curve, and any point that is in the intersection with the constant wavelength line indicates possible wave mode generation at that particular frequency range. The relationship is expressed as:

(13) $$C_{p}=f\lambda =fd$$

where λ is the wavelength of the desired ultrasonic wave.

## 3. Experiments and Analysis on Three Sensors

### 3.1. Cable Information

**Figure 2:**
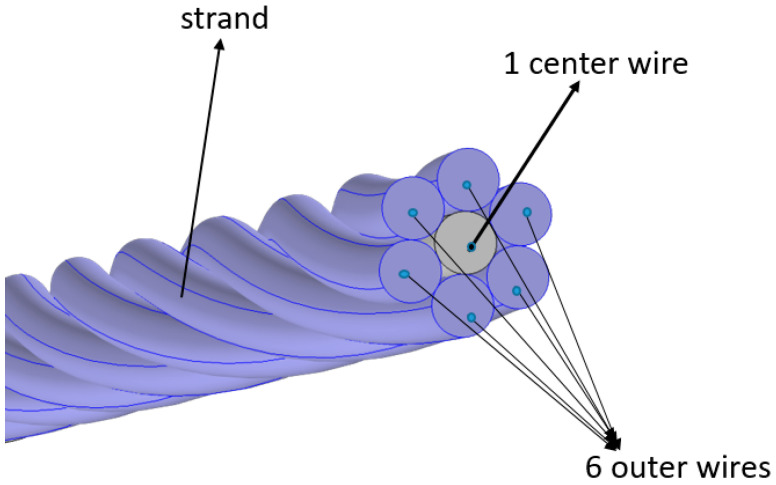
The configuration of the seven-wire steel strand used in these experiments.

The steel strand sample employed in this study is a 1.52 m-long seven-wire twisted steel strand with a defect. The model is shown in Figure 2 below.

The configuration clearly shows that the seven-wire steel strand has one single straight center wire, which is evenly surrounded by six twisted helical steel strands. To be more accurate, Table 1 shows more details.

**Figure 3:**
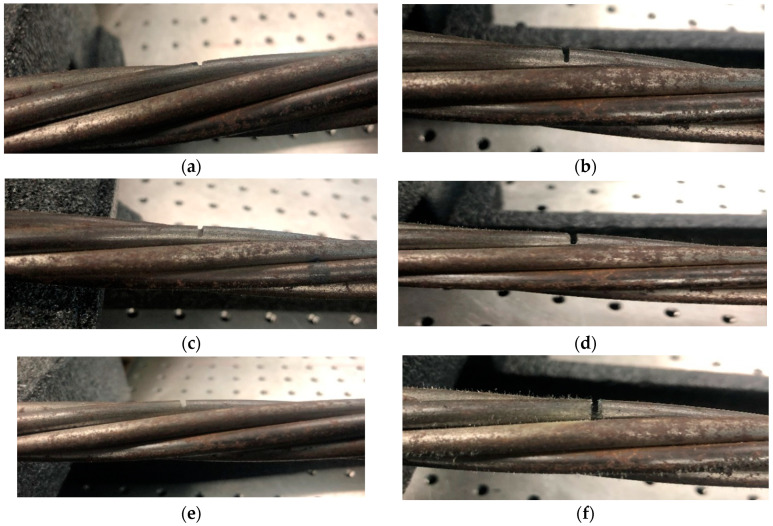
The seven-wire steel strands with different depth of defects for experiments (**a**) 1 mm, (**b**) 2 mm, (**c**) 3 mm, (**d**) 4 mm, (**e**) 5 mm, (**f**) 6 mm.

**Figure 4:**
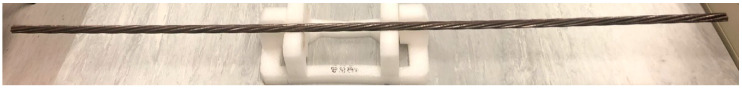
The whole view of the tested steel strand sample.

The seven-wire steel strand in this experiment has a gauge length of exactly 1524 mm. It is made of high grade 270 steel with Young’s modulus of 195 GPa, which belongs to a low relaxation type. The notch was machined to act as a small defect in exactly in the middle of the steel strand, and the other side of the free end of the steel strand is regarded as a large defect. To ensure the accuracy of the experiment, six groups of experiments were conducted in which the defects consisted of different depths (1–6 mm). The detailed characteristics of the steel strand defect and the overall view of the steel strand are shown in Figure 3 and Figure 4, respectively.

### 3.2. Dispersion Curves of a Seven-Wire Strand

**Figure 5:**
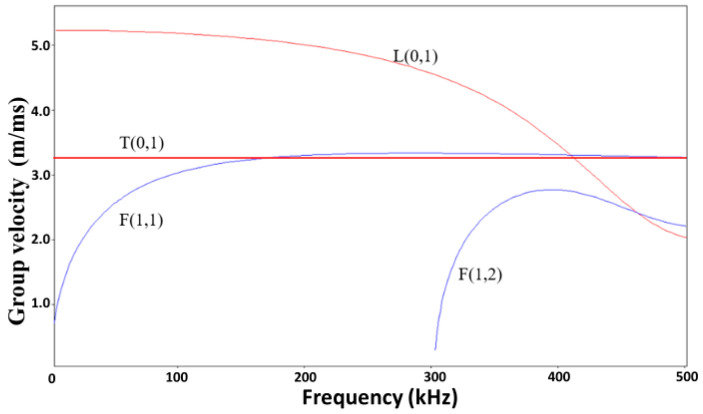
Group velocity dispersion curves of a steel straight wire with a diameter of 6.3 mm.

The propagation characteristics of UGW in the seven-wire steel strand can be obtained from its dispersion curves. Treyssède derived the dispersion curves by using the Semi-analytical finite element method (SAFE) and found there was almost no difference between the cylinder and helical wires wrapped around the steel strand when the lay angle is small [32,33]. The dispersion curve with group velocity and phase velocity profiles is validated almost no difference with previous research work. The dispersion curve was shown in Figure 5.

The frequency range of 100 to 300 kHz was selected as the excitation frequency region because the L (0, 1) mode was selected to excite the relatively optimized guided wave. The L (0, 1) mode was chosen because it is almost non-dispersive, and it travels much faster than other modes during the selected range. Therefore, it can be guaranteed that the receiver can receive the reflected wave signal firstly propagating in the L (0, 1) mode, and will not be confused with other modes propagating at a lower speed [34].

### 3.3. Experiments for Seven-Wire Twisted Steel Strand Defects Detection

Three different groups of experiments were conducted for detecting surface-defect in the steel strand. The first group was conducted with conventional MsS with a hard coil and later with Flexible Printed Coil (FPC). The result is presented in Section 3.3.1. The second group was conducted with a circular PZT that was attached to the surface of the end of the steel strand, and the result is presented in Section 3.3.2, while the description of the last group of Laser-based experiments is shown in Section 3.3.3. Additionally, the authors selected the 4 mm depth of defect as a representative to present the experimental results. However, for the results of other defects of different depth, the end of the experimental part could show them in a figure for comparison.

**Figure 6:**
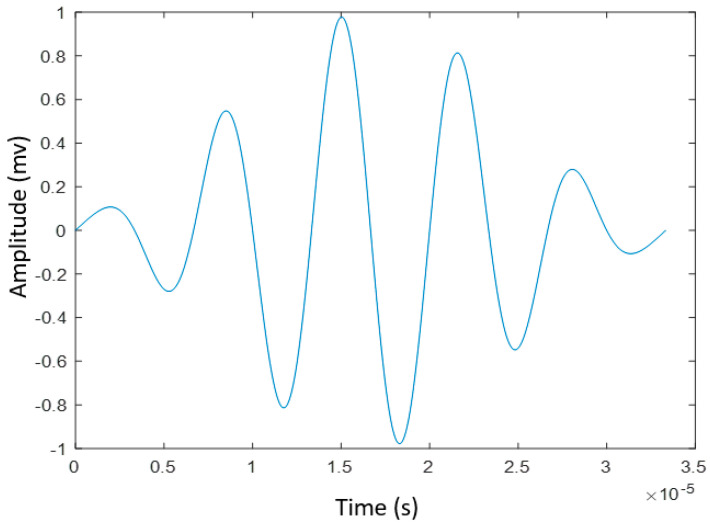
The five-cycle tone-burst excitation signal.

For the two groups of experiments with two types of MsS and PZT, a five-cycle tone burst signal was used as the excitation signal which is described as Equation (14) and Figure 6 below:

(14) $$S{\left(t\right)}_{excitation}=sin\left(\omega t+\theta \right)\left(0.08+0.46\left(1-cos(\frac{\omega t}{5})\right)\right)$$

where *t*, *ω*, and *θ* are the time, circular central frequency, and phase, respectively.

#### 3.3.1. Magnetostrictive Sensor (MsS)

**Figure 7:**
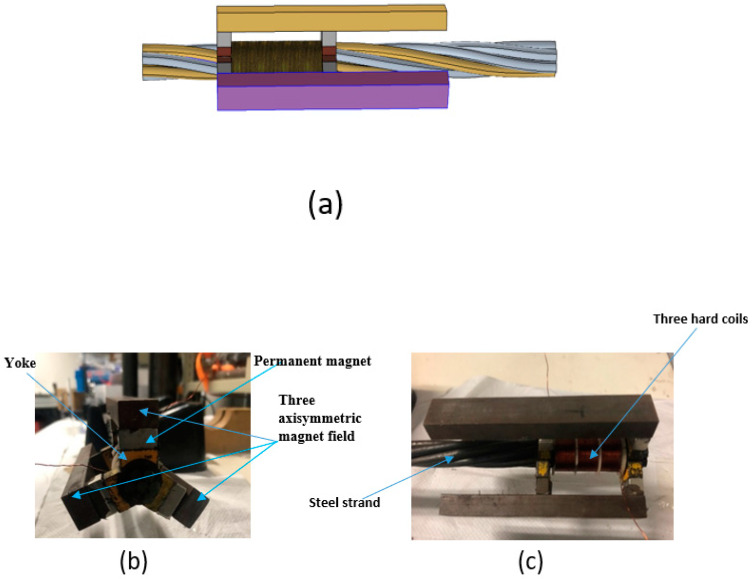
Schematic diagram of the hard coil magnetostrictive sensor (MsS): (**a**) the MsS model; (**b**,**c**) the components of MsS.

Considering the detecting stability and cost of MsS under actual engineering conditions, the same hard coil MsS correctly adopted and validated by Tse and Liu [34,35] has been used for surface defect inspection of seven-wire steel strands. Figure 7 shows the configuration of the hard coil MsS designed by Liu et al., which includes a sensing circuit with three axisymmetric magnets and yokes. The bias magnetic field generated by the permanent magnet is parallel to the dynamic magnetic field caused by the circuit of three sets of coils.

**Figure 8:**
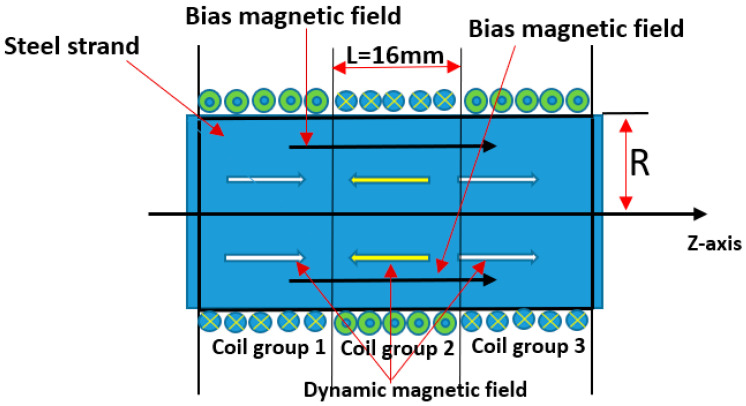
Arrangement of the hard coil MsS for excitation of the longitudinal guided waves in the steel strand.

As shown in Figure 8, this hard coil MsS consists of a permanent magnet circuit and an induction coil. When the dynamic magnetic field generated by the time-varying current flowing into the sensing coil is aligned with the magnetic field created by bias magnetic field, the steel strand will be mechanically deformed according to the magnetostrictive principle caused by the externally applied magnetic field, and therefore, the wave will propagate in the steel strand and cause the magnetic induction change of the induction coil according to the Villari effect. The expected L (0, 1) modes at group velocity of 5096 m/s and central frequency of 160 kHz were excited [34,35].

**Figure 9:**
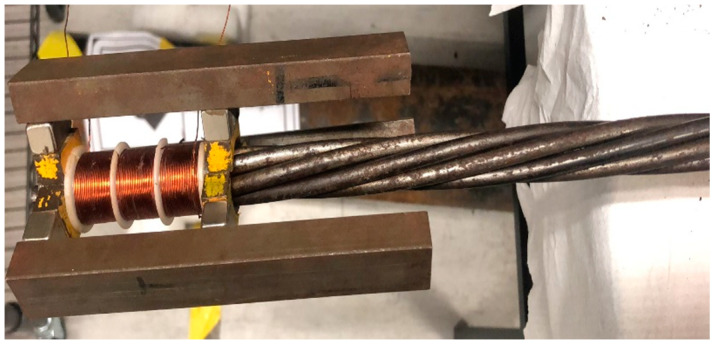
The real configuration of the hard coil Magnetostrictive Sensor (MsS) mounted to the end of the steel strand.

**Figure 10:**
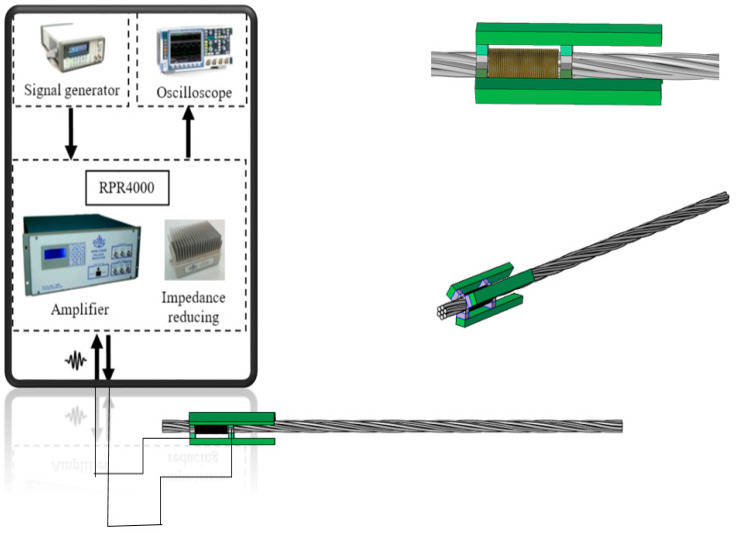
A specific illustration of the Experimental setup with hard coil MsS.

The hard coil used in the transducer had 31 turns with a length of 16 mm. The length of each group is equal to half of the wavelength of the excited guided wave mode. The first and third groups of the hard coil are wound in one direction, while the second group of hard coils was wound in the opposite direction. According to the group velocity dispersion curves, the half-wavelength of 16 mm is equal to the half-wavelength when the central frequency is 160 kHz and the group velocity is 5096 m s^−1^. The configuration and experimental setup of MsS are shown in Figure 9 and Figure 10.

**Figure 11:**
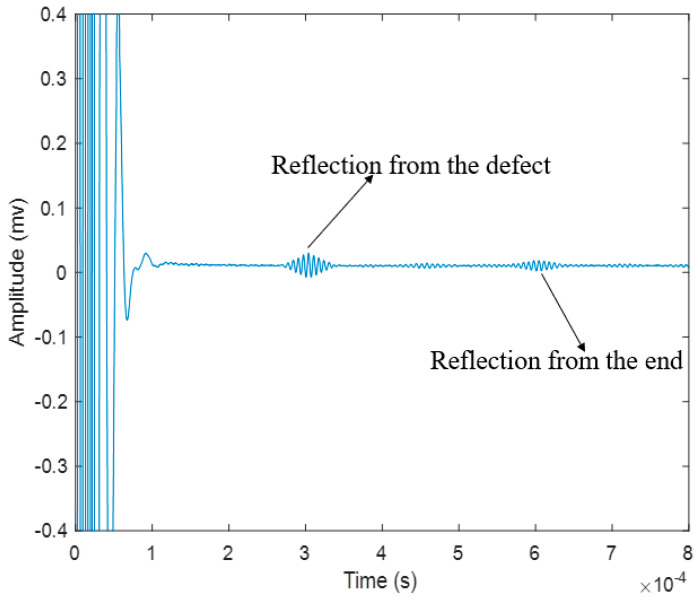
Signal of inspection for testing defective seven-wire steel strand with hard coil MsS.

As shown in Figure 10, the hard coil MsS is installed at the end of the steel strand, and the hard coil is inserted into the steel strand. The experiments were conducted with the pulse-echo mode. The excitation signal was first amplified by the Ritec RPR-4000 Pulse/Receiver and then went to the hard coils of the MsS. The best performance frequency of this type of MsS is around 160 kHz, and the testing result is shown in Figure 11.

In Figure 11, it could be easily found that the signal with traditional hard coil MsS is pure and the defect signal could be easily recognized.

According to the dispersion curves and Figure 10, the speed of L (0, 1) mode at 160 kHz is about 5096 m/s, and the reflection time of the defect and the end is around 0.304 ms and 0.609 ms, respectively, from the temporal waveform in MATLAB. At the same time, the theoretical time of defect reflection could be easily calculated:

(15) $$T_{reflection1\;}=\frac{x}{v_{1}}=\frac{1.524\;m}{5096\;m/s}=2.991\times 10^{-4}\;s$$

where *T_reflection_*_1_, *x*, and $v_{1}$ are the reflection time from the defect with traditional hard coil MsS, reflection distance from the defect, and the group velocity of L (0, 1) mode at 160 kHz, respectively.

**Figure 12:**
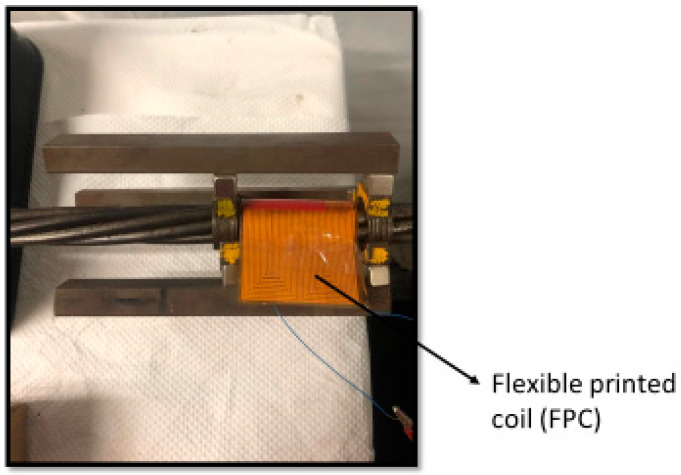
Experimental setup with the FPC-MsS that has three axisymmetric permanent magnets and the Flexible Printed Coil (FPC) to detect defects occurred in a seven-wire steel strand.

However, in actual field tests, the free end of the in-service steel strand is usually not accessible. Therefore, it is requested that some advanced coils that do not need to be inserted into the steel strand could replace the hard coil, which must have been inserted onto the steel strand. The flexible printed coil (FPC) can generate the required dynamic magnetic field, and the permanent magnet can trigger the required static magnetic field. The experimental setup is shown in Figure 12. This FPC was developed by Tse et al. [36], who obtained a patent. This is the first time this type of FPC is used in combination with permanent magnets to check the surface defects of the seven-wire steel strand.

**Figure 13:**
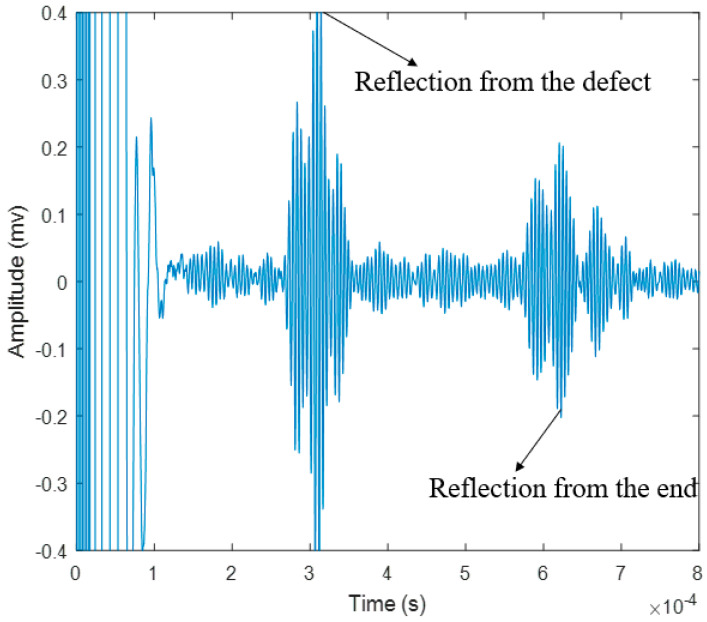
Reflected guided wave (GW) signals received by the FPC-MsS.

From the temporal waveform of the reflected GW signals, as shown in Figure 13, although some noise occurred during the reflection process, the defects and end signals of FPC-MsS are more obvious than those of hard coil MsS. The frequency of the best performance is around 200 kHz. According to the dispersion curves and Figure 13, the speed of L (0, 1) mode at 200 kHz is around 5027 m/s, and the reflection time of the defect and the end are around 0.309 ms and 0.619 ms from the temporal waveform on MATLAB respectively. At the same time, the theoretical time of defect reflection could be easily calculated:

(16) $$T_{reflection2}=\frac{x}{v_{2}}=\frac{1.524\;m}{5027\;m/s}=3.031\times 10^{-4}\;s$$

where *T_reflection_*_2_, *x*, and $v_{2}$ are the time of reflection from the defect with FPC-MsS, reflection distance from the defect, and the speed of L (0, 1) mode at 200 kHz, respectively.

#### 3.3.2. Piezoelectric Transducer (PZT)

**Figure 14:**
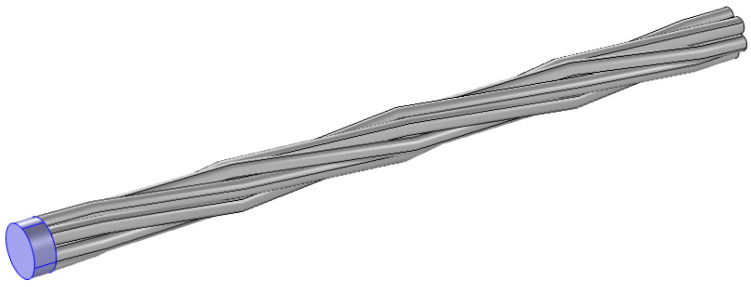
Configuration of PZT sensors attached to the seven-wire steel strands.

As shown in Figure 14, the circular PZT is installed on the end surface of the steel strand. For this research, these experiments were conducted by using a PZT to excite a five-cycle tone burst signal.

**Figure 15:**
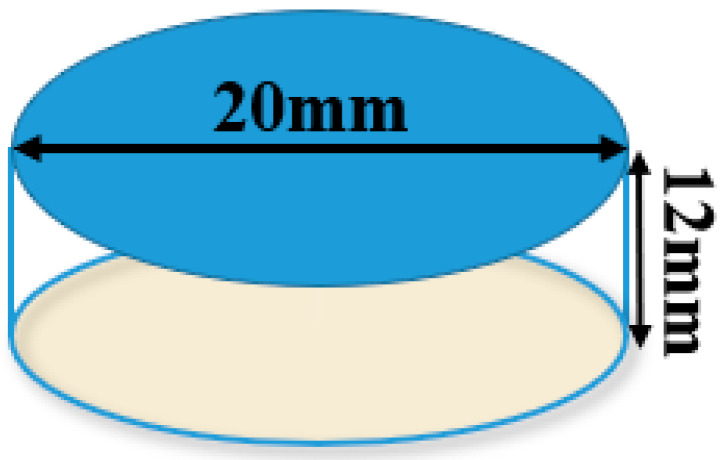
Specifications of circular PZT.

Figure 15 depicts a schematic diagram of the PZT structure, the diameter and thickness of which can generate the required guided wave mode. PZT is a device that utilizes the piezoelectric effect, which physically changes the shape by applying an external electric field on its two surfaces to generate ultrasonic guided waves. In this case, the PZT can be used to excite the longitudinal mode into the seven-wire steel strand under inspection. The tone burst is generated by the RITEC 4000 pulser and receiver (RITEC Inc., Warwick, RI, USA). A PZT transducer requires a couplant to excite and receive mechanical waves [37].

This type of PZT was manufactured from a ceramic manufacturing company and this PZT has a 20 mm diameter and 12 mm thickness. The central frequency of this PZT is 150 kHz and it belongs to SP-5 H disks.

**Figure 16:**
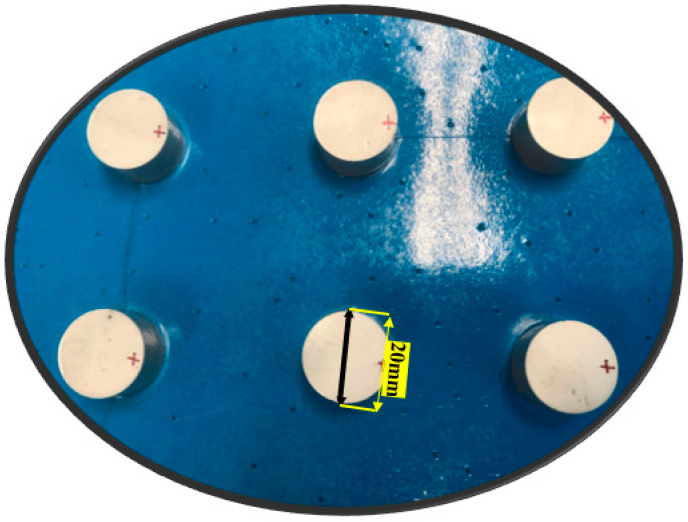
Configuration of PZT used in the experiment.

The actual configuration is shown in Figure 16.

**Figure 17:**
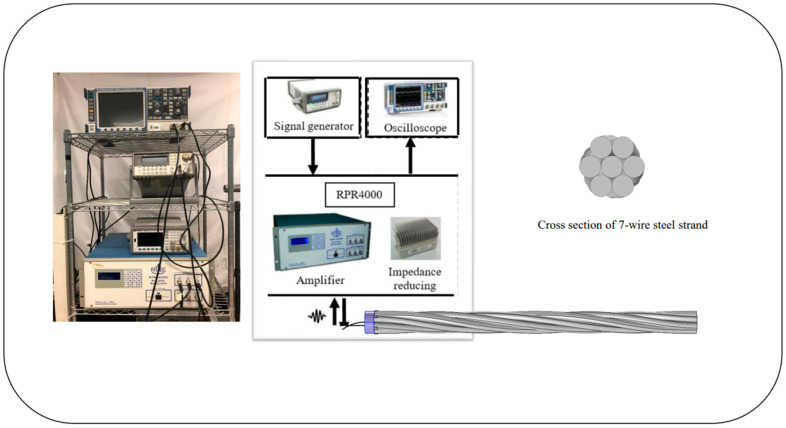
Schematic diagram of the experiment setup of PZT.

**Figure 18:**
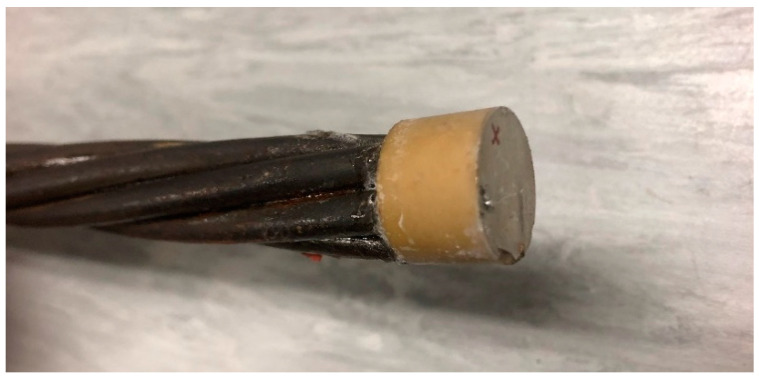
New circular PZT attached to the end of the strand by instant glue.

The experimental setup used to test the PZT capability is shown in Figure 17. Observed from Figure 17, the same equipment and mode were adopted as compared to that used to test the ability of MsS. The purpose of the comparison is to determine the experimental effect and eliminate the interference of other factors. The new circular PZT was made to cover the end of the steel strand and was mounted on the steel strand as shown in Figure 18.

**Figure 19:**
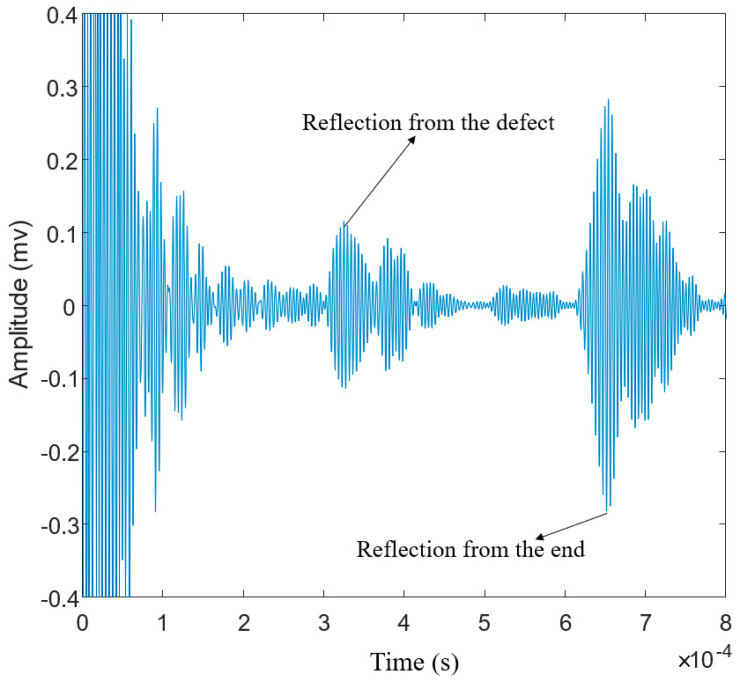
The signal received in pulse-echo mode from a defective strand using the new circular PZT sensor.

The experiment was carried out in pulse-echo mode at different frequencies. During the experiment, the frequency was adjusted by using 10 kHz step increments. The measured signal is shown in Figure 19. The frequency of the best performance is approximately 250 kHz. According to the dispersion curve and Figure 19, the speed of L (0, 1) mode at 250 kHz is about 4820 m/s, and the reflection time of the defect and the end are about 0.324 ms and 0.628 ms, respectively. At the same time, the theoretical time of defect reflection could be easily calculated:

(17) $$T_{reflection3}=\frac{x}{v_{3}}=\frac{1.524\;m}{4820\;m/s}=3.162\times 10^{-4}\;s$$

where *T_reflection3_*, *x*, and $v_{3}$ are the time of reflection from the defect with PZT, reflection distance from the defect, and the speed of L (0,1) mode at 250 kHz, respectively.

#### 3.3.3. Laser-Generated Guided Waves

**Figure 20:**
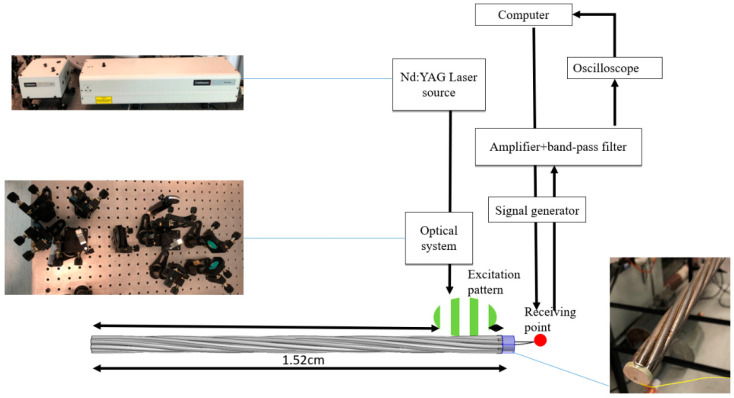
Experimental setup for noncontact laser-based GW system.

**Figure 21:**
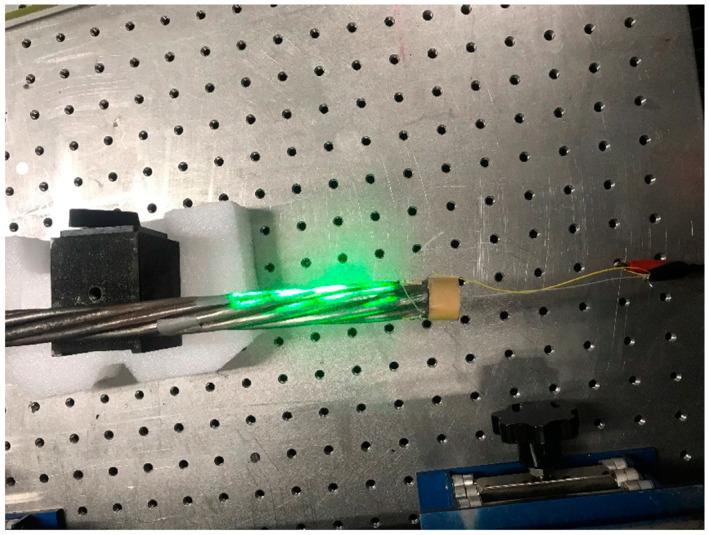
The laser hits the strand in the experiment.

The last group of experiments was conducted with the noncontact laser system. An Nd: YAG laser (SLIII-EX, Continuum Electro-Optics, Inc. Milpitas, CA, USA) with a wavelength of 532 nm was used as the laser source. The integrated Sagnac interferometer optical system [31,38,39] was used to spatially modulate the laser beam to generate a narrowband guided wave at the desired frequency range in the specimen. A PZT was attached at one end of the steel strand to act as the receiver. The received signal is displayed through an oscilloscope. The schematic of the experimental setup is shown in Figure 20. As shown in the figure, the pseudo-pulse-echo scheme was adopted. The laser excitation pattern was placed very close to the receiver as demonstrated in Figure 20 and Figure 21. The element width of the laser pattern is around 2.3 cm, and a total of four elements were created. Therefore, the pattern itself is 9.2 cm in width.

**Figure 22:**
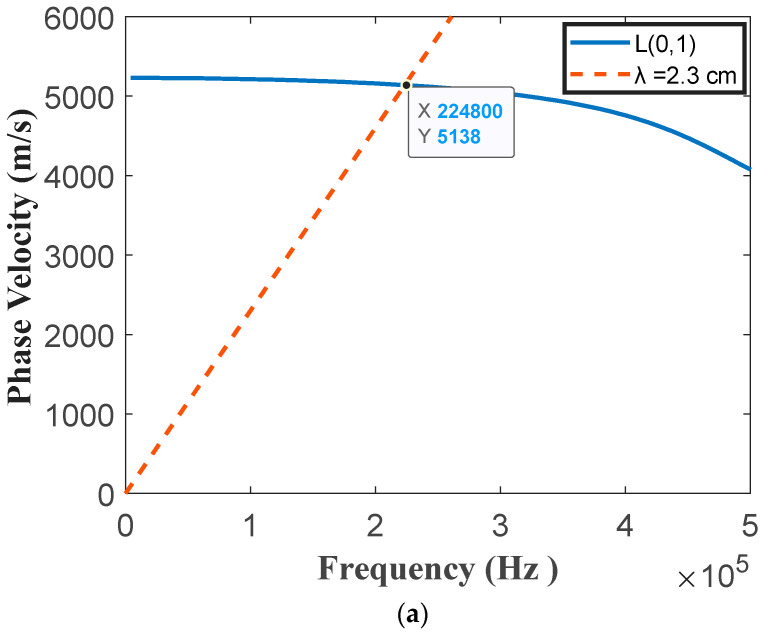

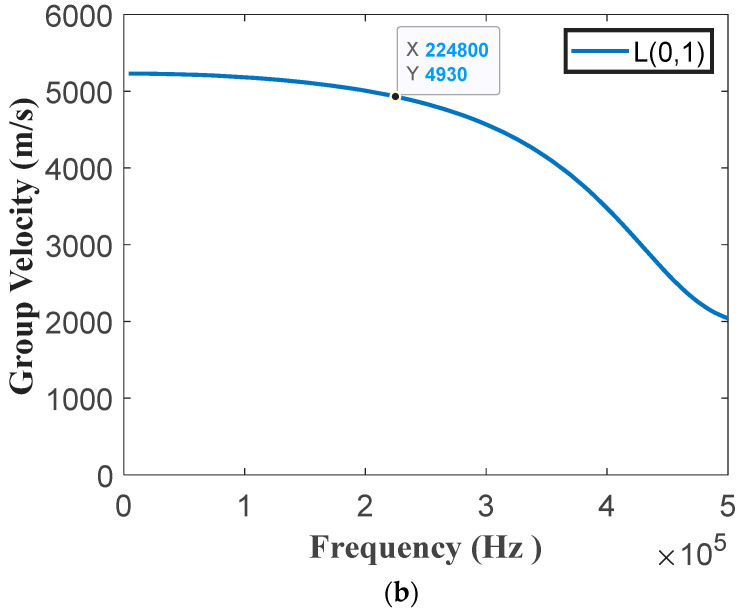
Dispersion curves of L (0, 1) in (**a**) phase velocity and (**b**) group velocity of the seven-wire steel strand.

From [31], the wavelength of the generated guided wave is equal to the element width of the laser pattern. The frequency of the laser-generated guided is determined by the following equation:

(18) $$C_{p}=f\cdot \lambda =f\cdot \Delta s$$

where *λ* is the wavelength of the generated wave, *∆s* is the element width, *f* is the frequency of the desired wave mode, and *C_p_* is the phase velocity of the desired wave at *f*. To determine the frequency, a constant wavelength line λ = 2.3 cm is plotted on the dispersion curve as shown in Figure 22. The point where the constant wavelength line intersects with the dispersion curve indicates possible wave mode generation. From Figure 22a, it is observed that there is an intersection point at around 225 kHz. This indicates that the L (0, 1) mode at 225 kHz will be generated. According to the dispersion curve in Figure 22b, the group velocity of the L (0, 1) mode propagates at around 4930 m/s.

**Figure 23:**
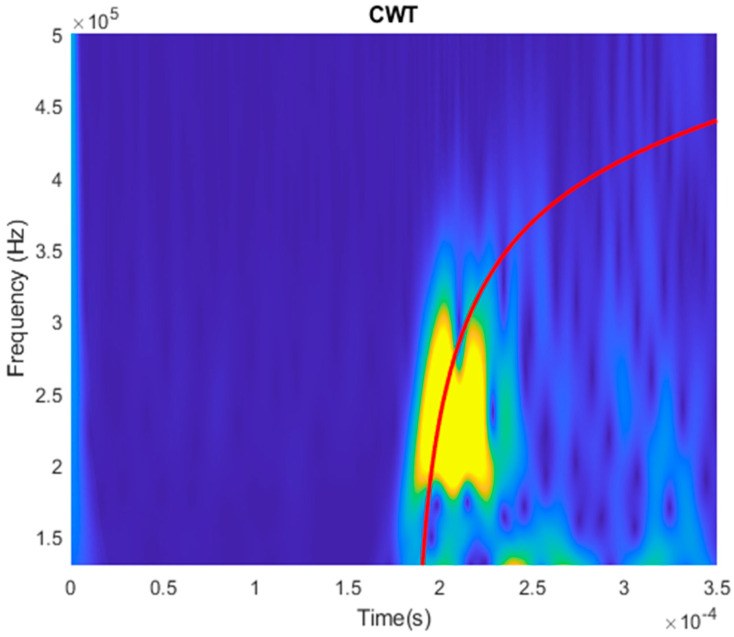
The time-frequency spectrum of a sample temporal plot to verify with the dispersion curve.

To verify that the guided wave was excited at the desired frequency range, the time-frequency spectrum of the received temporal signal was obtained and plotted in Figure 23. To ensure accurate determination, the excitation pattern was placed 980 mm away from the receiver. The dispersion curve of L (0, 1) was plotted on the time-frequency spectrum. As seen in Figure 23, the bandwidth of the generated wave mode is at around 200 to 300 kHz and the energy is concentrated at around 225 kHz. The generated wave mode matches the theoretical dispersion curve well. This shows that the wave mode was generated at the desired operating frequency. 

**Figure 24:**
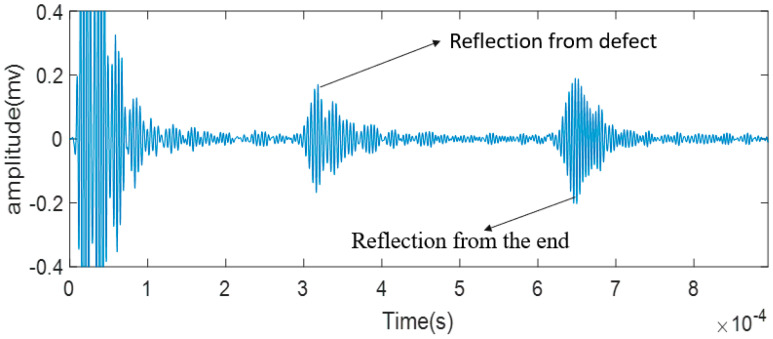
The received signal from the laser-guided wave system.

To study the effectiveness of using a laser-generated guided wave to assess the integrity of the strand, the pseudo-pulse-echo scheme as shown in Figure 20 was adopted. The defective specimen of the steel strand was used. The temporal signal was plotted in Figure 24. A bandpass filter from 200 kHz to 300 kHz was applied to obtain a better temporal plot. The expected arrival time of the reflected-defect signal is at 0.309 ms, whereas the expected arrival time of the end reflection is at 0.618 ms. From Figure 24, it is clear that two wave packets were captured. Their respective time of arrival is at around 0.319 ms and around 0.638 ms. The arrival time of the measured wave packets matches well with the expected arrival of the reflected-defect signal and the end reflection. This indicates that the defect is successfully located by using the laser-generated guided wave.

**Figure 25:**
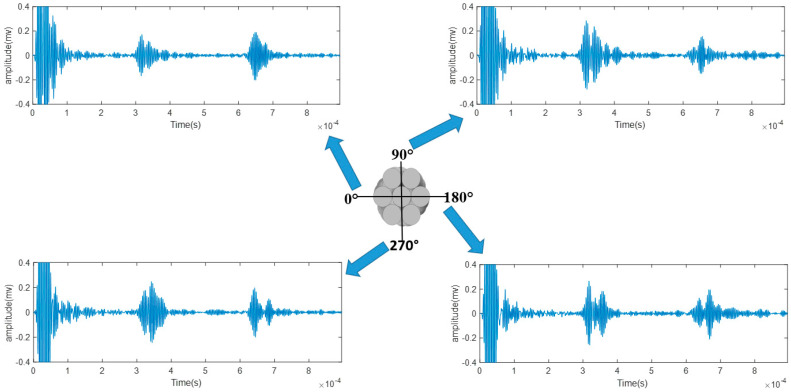
The received signal from different angles (0 degrees, 90 degrees, 180 degrees, and 270 degrees of seven-wire steel strand).

Since the laser only hit one side of the strand, it is worth investigating that whether rotating the strand would yield a different result. The temporal signals after rotating the strand for 90°, 180°, and 270° were obtained and are displayed in Figure 25. It is observed that there are the reflected-defect signal and the end reflection. All four measurements can successfully detect the defect regardless of the angle of rotation. Despite that, there are small fluctuations in the amplitudes of the wave packets. Overall, there is no obvious difference in the received signal.

Usually, the time interval of the peak-to-peak amplitude of the reflected wave packet is used to estimate the location of the defect. This is because upon interacting with the defect, a portion of the elastic wave energy is reflected and reaches the maximum when the peak of the incident wave hits the defect. Table 2 shows the arrival time and other characteristics of the defect reflection signal.

**Figure 26:**
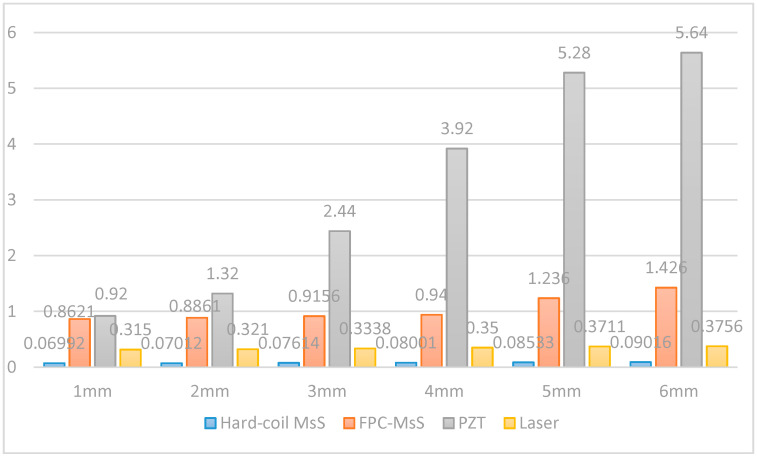
Variation trend of the amplitude of different sensor transduction systems for defects with different depths occurred in the steel strand.

For other groups with different depth of defects, Figure 26 shows the amplitude changing tendency for these four types of different sensor transduction systems on the different depth of defects.

#### 3.3.4. Comparison and Analysis of Results

From Table 2, it is obvious that that the four sensors can locate defects relatively accurately on the steel strand, but in terms of detection results, the temporal signal of traditional MsS with hard coils is particularly weak compared to the other three transduction methods. In terms of the waveform in the temporal signal, the PZT generated more noise/unwanted wave modes compared to the other three transduction methods as shown in Figure 11, Figure 13, Figure 19 and Figure 24. This may be because the coupling effect of instant glue is not very effective or the end surface of the steel strand is not polished very flat. The transduction energy, using PZT in this scenario, might be unevenly distributed, and thus, unwanted wave modes/noise were induced. However, the transduction power of PZT is relatively stronger as shown in Figure 26. It is more sensitive to the change of defect, as can be seen in Figure 26, where PZT has the steepest slope comparatively.

Compared with PZT and laser-based GW systems, the signal of the hard coil MsS is relatively easier to identify and it could trigger a single L (0, 1) mode as can be seen in Figure 11. Thus, its defect can be precisely located during the on-site test. However, for the long-inspection on-site test, hard coil MsS may not be favored because the energy generated by this type of sensor is quite low and there may leakage of energy during the detection process due to several mechanical and electrical energy conversions.

Low-cost PZT patches can be easily integrated with various structures through surface bonding, and have been widely used in vibration detection, vibration control, shape control, energy harvesting, stress wave generation, damage detection, and structural health monitoring [40]. However, for the case of using PZT to inspect the twisted steel strand, the free end of the strand cannot be easily accessible, which greatly hinders the feasibility of using PZT system as transduction means to inspect the integrity of the strand on site. Moreover, the PZT Patch is difficult to attach to the surface of the strand because the single wire of the strand is curved and the PZT must be evenly distributed along the circumference. Thus, the open free end surface of the steel strand attached by circular-shaped PZT is the best option. Additionally, when we mount it to the free end surface of the steel strand, the surface of the open free end surface is smoother than the other surfaces of the steel strand, which can be used to trigger guided waves more uniformly.

For small defects, such as 1–2 mm defects that occurred on the steel strand, the capabilities of these four sensors could detect it easily without changing too much amplitude, except PZT, which caused a relatively large change of amplitude. For relative bigger defects (such as 3–6 mm depth of defects), laser-based guided wave system- and hard coil MsS could detect it without changing the amplitude of the signal too much with the increment of the depth of defects. However, the PZT and FPC-MsS could also detect it easily but the signal amplitude will vary greatly as the depth increases, especially for FPC-MsS. It could be easily concluded that the FPC-MsS and PZT are more sensitive to the larger defects with greater depth. In contrast, the hard coil MsS and Laser-based GW system have weaker capabilities to detect defects and are less sensitive to changes in the depth of defects.

It is worth noticing that, in a practical environment, there are possible artifacts that can be introduced into the measurement by different methods. For example, when there is a defect very close to the end of the steel strand to which the PZT is attached, it will be obscured by the direct reflection of the UGW. The defects may not be detected in this range, because the generated power of UGW here is huge and may obscure the reflection signal of the defect. Moreover, if the defect occurs at the end of the steel strand, the reflection of the defect may overlap with the end reflection. In fact, this is a very common scenario, which is usually named the ‘dead zone’ that always occurs [41,42,43,44,45,46,47] in the contacting NDT methods, because these methods need some part of the sensor to be in contact with the steel strand to evaluate the defected objects, and the power of UGW generated here always be larger than that of the area of the reflection wave. However, non-contact NDT methods, such as laser-based GW system, do not require any sensors to be mounted on the surface of the inspected objects; if the ultrasonic power generated by the laser-based GW system is sufficient, it can detect any area of the object.

## 4. Results and Discussion

Because the purpose of this study is to provide some practical suggestions for the inspection of seven-wire steel strands under actual engineering conditions, especially in the pre-installation state, post-installation state, and in-service states such as steel strands assembled in bridges, elevators, ground anchors, buildings, water tanks, and other areas, we recommend that the health state of the seven-wire steel strand is checked timely and accurately to ensure the safety of the entire structures.

From an economic point of view, compared with the other two methods, laser-based experimental instruments are obviously the most expensive ones, which generally cost several million Hong Kong dollars. Based on the cost of professionals and maintenance personnel, the laser-based system also needs a large investment to train the workers for a long time for this type of work compared with the expenditure of the other two methods. As for the various costs of MsS and PZT for field testing, the cost of MsS is almost the same as that of PZT, and the price of a single PZT or FPC with relatively good quality ranges from several hundred to one thousand Hong Kong dollars, while the hard coil MsS cost is lower than other two types of sensors. However, in terms of staff costs and maintenance costs, the cost of MsS should be higher than PZT, because the workers only need to mount a single circular PZT on the end surface of the steel strand, but need to mount the permanent magnet, hard coils or FPC, and other related components on the seven-wire steel strand.

From a technical point of view, it is obvious that the laser system requires the most professional personnel to perform the most precise operation. This is because the operation of high energy laser can be damaging to eyes and skin and requires special care. Comparatively, the operation of the MsS is simpler, because for the installation of the sensor, personnel hardly needs to pay special attention to its reliable and safe operation. The relative position of the permanent magnet and the coil or FPC should be correct and the dynamic magnetic field and static magnetic field should be matched. On the contrary, in terms of the effectiveness and feasibility of the detection method, it is most difficult to guarantee the detection efficiency of the laser system, because its excitation frequency cannot be precisely controlled. At the same time, prisms and other reflective systems require a lot of preliminary adjustments; the ease of use of MsS and PZT is almost the same, but for MsS, due to the small energy transmission and short detection distance, the installation of sensors requires a relatively more complicated process compared to installing PZT. Therefore, PZT is considered to have the following advantages: it is a simple and proper effective steel strand Line detection method. Unlike the electromagnetic acoustic transducer (EMAT), manufacturing PZT requires special equipment and technology, which means that its parameters are difficult to study. At the same time, because PZT has a high signal-to-noise ratio, it has been commercialized already. The middle layer and liner layer are added to the PZT to change its frequency and amplitude characteristics. Moreover, the PZT company can manufacture PZT according to customer needs. However, the manufacturing process lasts for several weeks generally. Moreover, PZT is neither cheap nor fragile compared to hard coil MsS. For these reasons, research on PZT triggering GW is not cheap, while being time-consuming to some extent.

Finally, considering the stability of the test instrument, the detectable distance, and range, the laser-based GW system is definitely the best option, with a detection distance ranging from 125 mm to 100 m [48], and once the laser-based GW system equipment is properly prepared, it is comparable to PZT and MsS. In comparison, the frequency and other characteristics of the laser-based guided wave it emits are stable for a long period. Moreover, the power of PZT is greater than that of MsS with hard coils, thus, the inspection distance of PZT is longer, and because the position of the MsS component needs to be adjusted occasionally to ensure that the guided waves excited by it are the same, PZT seems to have better inspection stability than MsS.

According to the experimental results and the above analysis, the following table lists the five most important factors in actual engineering projects. The performance indicators of these three types of sensors were evaluated objectively and fairly, and the results are listed in Table 3.

It can be easily found from Table 3 that the FPC-MsS is outstanding in terms of economy. Among the three NDT methods, the laser-based GW system has high inspection stability and the longest detection distance and range, but it needs higher investment to ensure this system functions well when compared to other sensor systems. MsS is easier to be used as a promising tool for short-distance inspection and some engineering project which need an accurate inspection, and especially the FPC-MsS can be used to inspect those steel strands without free ends. Compared with the other two methods, the PZT may be suitable for relative long-distance inspection. However, different companies may consider different requirements or inspection levels for specific inspection tasks or projects.

## 5. Conclusions

The effectiveness of the three NDT methods on the inspection of seven-wire twisted steel strands was experimentally investigated. In short, for on-site testing, a laser-based GW system is more suitable for indoor inspection. Especially when the steel strands need to be inspected before inspection in a clean warehouse. As a non-contact system that can be functional in narrowband and broadband types of excitation, it is suitable for recording large amounts of data to analyze guided wave behavior in more depth and it could have stable detection ability for defects of different depths on steel strands. MsS, which is usually used in the narrowband range, is the proper tool for pinpointing defects. GW signals recorded by MsS especially hard coil MsS are generally very clean with minimal noise and contamination. However, the hard coil MsS generate less power than other types of the sensor, which could to some extent limit their ability of long-distance inspection, and its sensitivity to defects of different depths does not change much. In contrast, FPC-MsS is more sensitive to defects of different depths and has a better ability to inspect longer distances than hard coil MsS. MsS, that is also a convenient method that can be installed on steel strands without having to access their free ends. Additionally, it is more cost-effective than other transduction systems and is easy to carry around for field testing. The circular PZT can be easily mounted on free ends of steel strands. Compared with MsS, it generates much stronger signals, which is beneficial for long-distance inspection. PZT has obvious gradation in the detection sensitivity of different depth defects, which makes it more suitable for the detection of large depth defects. It may also be more advantageous if there is a concern of higher attenuation as a result of contacting steel strands with other materials such as concrete. However, the installation of the circular PZT would require access to the free end of the steel strand, making it less favorable for many actual cases on-site.

In addition, based on economic and technical considerations, a simplified evaluation framework is proposed, which is used for selecting appropriate NDT methods to inspect a healthy state of steel strands. In the analysis and discussion section, some specific suggestions are put forward to check the surface defects of steel strands when facing different actual environments. Relevant companies or engineers can choose practical and effective NDT methods to inspect the healthy state of seven-wire twisted steel strand based on the evaluation framework proposed in this article and the recommendations provided above.

## Figures and Tables

**Table 1:** Specifications of seven-wire steel strands used in this experiment.

Nominal Diameter (mm)	Diameter Tolerance (mm)	Grade (MPa)	Nominal Weight (g/m)	Nominal Steel Area (mm^2^)	Minimum Breaking Strength (kN)	Yield Strength Minimum Load at 1% Extension (kN)
15.24	–0.15/+0.65	1860	1102	140.0	260.7	234.6

**Table 2:** Evaluation framework of typical Non-Destructive Testing (NDT) methods for surface defects inspection on steel strand.

NDT Methods	Time Arrival of Defect-Reflected Signal (Peak to Peak Amplitude) (s)	Estimated Defect Location (m)	Deviation from Actual Location (%)
Hard coil MsS	$3.038\times 10^{-4}$	0.7741	1.59
FPC-MsS	$3.092\times 10^{-4}$	0.7771	1.99
PZT	$3.244\times 10^{-4}$	0.7818	2.60
Laser-based GW	$3.190\times 10^{-4}$	0.7863	3.19

**Table 3:** Evaluation framework of typical NDT methods for surface defects inspection on steel strand.

	NDT Method	FPC-MsS	PZT	Laser-Based GW System
Evaluation Index				
Feasibility of NDT methods available		H	M	L
Cost of equipment and Related maintenance or employee costs		L	M	H
Testing instrument stability, detectable distance, and range		L	M	H
Requirements regarding reliable and safe operation		M	L	H
The accuracy of defect location		H	M	L

The three capital letters ‘L, M, H’ represent the ‘Low, medium, high,’ respectively, which represent the different levels on each evaluation index from low to high only among these three NDT methods.
